# Spotted Fever Rickettsiosis in a Wildlife Researcher in Sabah, Malaysia: A Case Study

**DOI:** 10.3390/tropicalmed3010029

**Published:** 2018-03-06

**Authors:** Milena Salgado Lynn, Timothy William, Ampai Tanganuchitcharnchai, Suthatip Jintaworn, Janjira Thaipadungpanit, Mei Ho Lee, Cyrlen Jalius, Peter Daszak, Benoît Goossens, Tom Hughes, Stuart D. Blacksell

**Affiliations:** 1 Danau Girang Field Centre, c/o Sabah Wildlife Department, Sandakan 90009, Sabah, Malaysia; Salgado-LynnM@cardiff.ac.uk (M.S.L.); fielondra1988@gmail.com (C.J.); GoossensBR@cardiff.ac.uk (B.G.); 2 School of Biosciences, Cardiff University, Cardiff CF10 3AX, UK; 3 Wildlife Health, Genetic and Forensic Laboratory, c/o Sabah Wildlife Department, Kota Kinabalu 88100, Sabah, Malaysia; 4 Sustainable Places Research Institute, Cardiff University, Cardiff CF10 3BA, UK; 5 Jesselton Medical Centre, Kota Kinabalu, Sabah 88300, Malaysia; tim7008@gmail.com; 6 Centre for Tropical Medicine and Global Health, Nuffield Department of Clinical Medicine, University of Oxford, Churchill Hospital, Oxford OX3 7FZ, UK; ampai@tropmedres.ac; 7 Mahidol-Oxford Tropical Medicine Research Unit, Faculty of Tropical Medicine, Mahidol University, Bangkok 10400, Thailand; suthatip@tropmedres.ac (S.J.); Janjira@tropmedres.ac (J.T.); 8 EcoHealth Alliance, New York, NY 10001-2320, USA; Lee@ecohealthalliance.org (M.H.L.); daszak@ecohealthalliance.org (P.D.); tom.hughes@ecohealthalliance.org (T.H.); 9 Sabah Wildlife Department, Kota Kinabalu, Sabah 88100, Malaysia

**Keywords:** rickettsiae, Malaysia, diagnosis

## Abstract

We present evidence for a case of spotted fever rickettsiosis with severe complications in a young adult male. Although spotted fever group rickettsiae (SFGR) have been reported as the most prevalent cause of rickettsiosis in rural areas of Sabah, Malaysia since the 1980s, this is the first detailed case report of suspected SFGR in the state. Current data on the prevalence, type, and thorough clinical reports on complications of SFGR and other rickettsioses in Sabah is lacking and required to raise the awareness of such diseases. There is a need to emphasize the screening of rickettsioses to medical personnel and to encourage the use of appropriate antibiotics as early treatment for nonspecific febrile illnesses in this region. Suspected rickettsioses need to be considered as one of the differential diagnoses for patients presenting with acute febrile illness for laboratory investigations, and early treatment instituted.

## 1. Introduction

Over the past 25 years, there has been an increase in the knowledge of rickettsial pathogens worldwide [1]. Nevertheless, there are still geographical areas where little information on rickettsioses is available regarding the clinical cases, the pathogens and the vectors [1,2,3,4]. Yet in Southeast Asia, and in travellers returning from this region, rickettsial diseases are among the leading causes of treatable acute febrile illnesses [5,6]. The clinical symptoms of rickettsioses may vary depending on the rickettsial species involved, and therefore diagnosis can be challenging even for experienced physicians [1]. Moreover, the specific laboratory methods for the diagnosis of infections are seldom available in remote areas, and even in large cities, in Southeast Asia [1,5,7]. In addition, the management of the disease could also be challenging given the (lack of) availability of the most commonly used antibiotics, the possible resistance to them, and the alternatives to such antibiotics [8,9].

Rickettsioses are considered endemic in Malaysia and mostly associated with rural/estate areas [10,11] though current data on the prevalence and type of rickettsioses are scarce [12,13,14,15,16]. Reports of this disease in Sabah are limited to two retrospective serological studies in the early 2000s [13,14], one cross-sectional survey in 1986 [17], and one prospective study in 2017 [18]. In this report, we present evidence for a case of spotted fever rickettsioses in a wildlife researcher in Sabah, Malaysia, highlighting the need for increased awareness of rickettsioses as a cause of acute febrile illness in this region.

## 2. Clinical History

**Figure 1:**
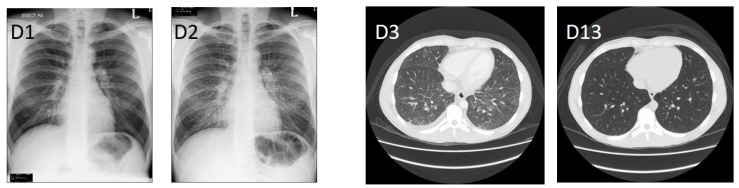
Chest X-rays upon admission (D1) and subsequent day (D2). Contrast-enhanced CT scan after oral and intravenous contrast administration at the day of hypotensive shock (D3), and post-treatment (D13).

A previously-healthy 23-year-old British male presented to Jesselton Medical Centre (JMC), Kota Kinabalu, Sabah, Malaysia, with an acute febrile illness. The patient had been in Sabah for a period of three months prior to becoming ill. Earlier on the day of admittance at JMC the patient had attended a clinic from where he was discharged to wait two days for the results of diagnostic tests (dengue and malaria), evidently not recognising the severity of his symptoms. Upon admission to JMC (day 1, D1), the patient reported onset of symptoms four days prior, having high fever (over 39 °C), headache, dry cough, sweating, diarrhoea, joint and muscular pain, appetite loss, nausea, confusion and light-headedness. Physical examination indicated a mild rash of small, undefined, scattered red dots on the limbs and abdomen and mild haemoptysis, at which point he was admitted to the intensive care unit (ICU). Haematology and biochemistry results indicated severe thrombocytopenia, leukopenia, lymphopenia, and a mild cholestasis (Table 1). He was haemodynamically stable, his lungs were clear, and there was no evident hepatosplenomegaly. Following admission, the patient’s condition rapidly deteriorated. The admission chest X-ray was normal, but a repeat X-ray the following day showed clear pleural effusion (Figure 1). On D3 post-admission, the patient went into hypotensive shock (BP 70-80/30-40). CT scans showed pleural effusion in the right lung as well as ground-glass opacities in the dependent portion of the lungs, bilaterally (Figure 1). Additionally, the liver and the spleen were enlarged at 21 and 15 cm, respectively. Signs of abnormal liver function became evident on D5 post-admission and were persistent for more than two weeks (Table 1). Antibiotic therapy commenced with ceftriaxone 2 g o.d. and doxycycline 100 mg b.d., with the former discontinued after the hypotensive shock on D3 post-admission, and the latter on D7 post-admission. Due to the patient’s inability to take oral medication, the lack of diagnosis, and to ensure a broad coverage of illnesses, meropenem 1 g tds and azithromycin IV 500 mg o.d. were started on D3 and D4 post-admission for four and seven days respectively; intravenous doxycycline, the preferred choice for this case, is not available in Malaysia. The patient became afebrile on D5 post-admission and subsequently improved over the following 2 weeks. He was discharged after 23 days hospitalization when he was fit to travel to his home country, where he made a full recovery.

Prior to admission, the patient had been working in the forests of the Sandakan Division in Sabah for 20 days to perform ecological research in both urban and rural environments. At the field camps, he reported being in close proximity to cats and dogs, and occasionally with rats, and denied direct contact with wild vertebrates. The patient had multiple insect bites, including presumptive mosquito bites, but no tick bites, even though the patient and his team reported a large number of ticks in the last forest that was visited one week prior to the onset of the fever. Although attired in designated clothing for fieldwork, garments were not always immediately removed upon arrival at the base camps.

## 3. Laboratory Diagnosis

Initial laboratory investigations at JMC included dengue (IgM antibody and NS1 antigen), malaria (blood film), leptospirosis (IgM antibody and IgG antibody), and influenza A and B (virus A and virus B antigens). All tests were negative. A multiplex PCR for 33 respiratory viruses and bacteria (FTD respiratory pathogens 33; Fast-Track Diagnostics) was positive for rhinovirus and also for influenza (A–F). An added tropical fever panel multiplex PCR (FTD Tropical Fever Core; Fast-Track Diagnostics) that included seven viral and bacterial families including *Rickettsia* spp. (targeting the citrate synthase gene, gltA) gave negative results for all pathogens. The multiplex PCRs were outsourced by JMC to a laboratory in West Malaysia. Additional throat, nasal, and rectal swabs, and urine were collected for PCRs, at the Sabah Wildlife Department’s Wildlife Health, Genetic and Forensic Laboratory, on pooled cDNAs, screening for coronavirus [19,20], filovirus [21], influenza [22], paramyxovirus [23], enterovirus ([24], unpublished; VP4/2 and 5′UTR genes, Centre for Infection and Immunity, Columbia University, 2013), flavivirus [25], and hantavirus [26,27] using the PREDICT Universal Control. A PCR product was obtained from the enterovirus protocol [24] and confirmed by sequencing to be a 98.6% match to human rhinovirus A30 HRV30/DJ0692/11 (KC414928, 513 bp), which did not explain the patient’s symptoms.

**Figure 2:**
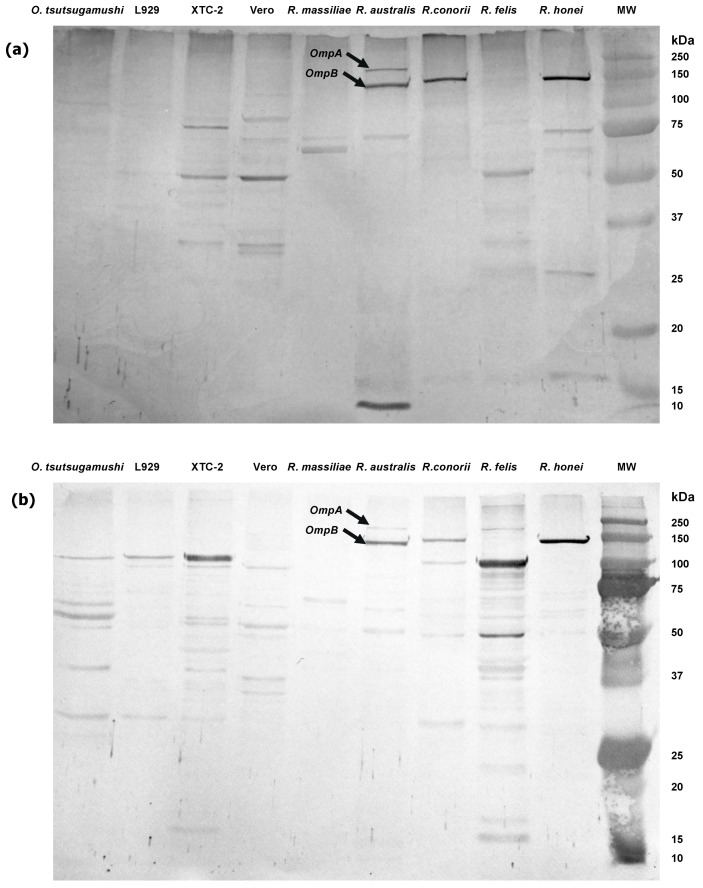
Western blot of the patient’s serum reacted at a 1:80 dilution against host cell cultures and *Orientia* and *Rickettsia* spp. antigens using (**a**) IgM and (**b**) IgG conjugates. L929, Vero and XTC-2 are continuous cell lines used to propagate *R. massiliae* (Vero), *R. conorii* (L929), *R. felis* (XTC-2), *R. honei* (L929), *R. australis* (Vero) and *Orientia tsutsugamushi* (L929). MW = molecular weight marker in kiloDaltons (kDa).

Rickettsiosis was suspected due to the patients’ response to doxycycline and azithromycin therapy. Samples were tested retrospectively for genetic and serological evidence of rickettsial infection. Unfortunately, there was only a limited number of samples available for testing as the admission blood and serum samples were destroyed on D7. Additional samples were collected on D15 for PCR and serology testing. A real-time PCR for scrub typhus group (STG-*Orientia tsutsugamushi*), typhus group (TG—*Rickettsia typhi* and *R. prowazekii*) and SFGR (*Rickettsia* spp.) were all negative. An indirect micro-immunofluorescence assay (IFA) against STG (*Orientia tsutsugamushi* Karp, Kato and Gillam strains), TG (*R. typhi* Wilmington strain) and SFGR (*R. honei* and *R. conorii*) antigens was performed, with only SFGR positive (IgM 1:80, IgG 1:10). Serological examination of SFGR members *R. honei* (IgM 1:40, IgG 1:20), *R. australis* (IgM 1:40, IgG 1:40), *R. conorii* (IgM 1:40, IgG 1:20), *R. felis* (IgM 1:20, IgG 1:10) and *R. massiliae* (IgM 1:20, IgG 1:20), confirmed the result and indicated cross-reaction amongst the SFGR species. IFA results were confirmed by western blot (WB) [28] that demonstrated specific *OmpA* (130–160 kDa) and *OmpB* (115–150 kDa) bands for *R. honei*, *R. australis* and *R. conorii*, when reacted with the patient’s serum using IgM and IgG conjugates (Figure 2).

## 4. Discussion

Based on the available evidence of response to doxycycline and azithromycin therapy and on the seropositivity demonstrated via IFA and WB tests against SFGR, there is evidence suggestive of SFG rickettsiosis in this patient. Since the patient was from a non-SFGR endemic region the relatively modest IFA titers may be consistent with a primary infection. Given that the sample was collected 15 days post-admission to hospital and 20 days post-onset of fever it was unlikely that the sample would be PCR positive. However, the PCR performed on the admission sample was negative, probably due to the lack of a whole blood pre-culturing step recommended by the manufacturer when typhoid is suspected. Therefore, the multiplex format performed might have lacked the sensitivity this case required. Also, the large variability of species belonging to the genus *Rickettsia*, and those yet to be identified in this region, might be another possibility [1,5]. The lateness of the sample collection and lack of early and follow-up samples is a weakness in this case study as there was no opportunity to demonstrate quantitative rise in antibody titer or to demonstrate the infectious agent via PCR.

Sabah and other similar areas of rural Malaysia are considered biodiversity hotspots and growing ecotourism destinations. There is greater involvement in outdoor activities in rural and/or remote areas, which may increase the contact with vector-borne pathogens. Additionally, shifts in land-use and deforestation have increased dramatically in Sabah during the last decades [29]. Combined, all these characteristics can be relevant to disease (re-)emergence, especially *Rickettsia* spp. For Sabah, the retrospective studies focused only on the health centre of Nabawan province of the Interior Division (rural), and on patients attending Queen Elizabeth Hospital (QEH) in the state capital (urban). The study of Tay et al. (2000), reported highest rickettsial seropositivity in Nabawan (91.7%; *n* = 145) where 84.8% were SFGR (*R. honei*, then TT118 strain) and 40% of the seropositive patients were agricultural workers. In another Sabah study, seven rural villages (*n* = 1220) in the Tawau and Kudat Divisions [17] demonstrated SFGR seropositivity four times higher in the two forest-dwelling populations of the Tawau Division (16.5% each). Knowledge on the vectors and non-human hosts of rickettsial illnesses in Sabah is also limited. Ticks from rats collected in an island of the north of Sabah were all negative to SFGR by PCR [30]. However, 74 % of fleas collected from domestic dogs in Sabah’s West Coast Division were all positive to *R. felis* qPCR [31].

The diagnosis and treatment of rickettsioses in Sabah are highly challenging. First, rickettsial infections are probably more common than currently accepted. In 2015, the Malaysian Ministry of Health (MMoH) reported only one case of typhoid fever nationwide [32]. However, the lack of routine testing capacity means formal MMoH notifications likely underestimate the true case incidence of rickettsial illnesses. For instance, a recently completed prospective study in northern Sabah demonstrates the common occurrence of rickettsioses among those with non-malarial acute febrile illness with an adjusted incidence of 6.5 cases/million for this state (a 23-fold increase from MMoH estimates) [18]. Second, in a region where dengue, malaria and leptospirosis (to name a few) are present, the lack of presence of a rash and/or an eschar can easily confound a physician who is not aware of rickettsiosis present in the area. Serology, in particular (micro)immunofluorescence assay, remains a gold standard for diagnosis, even when it has already been suggested that this technique should only be considered in areas with previously established seroprevalence [1,33], something that has not been done in Sabah. Moreover, the experienced manpower needed to perform the tests and to minimize the misinterpretation of results is, in most cases, lacking. In addition, specific equipment is also needed and it is often unavailable in the rural/estate areas, where these diseases are more likely to be present in Sabah. Nucleic acid methods present the same limitations as serology in such regions. Even in urban areas, serology may be adequate for diagnosis of spotted fever rickettsiosis, but the etiologic agent is not likely to be identified [1]. To reduce the delay in diagnosis, a rapid antigen-based diagnostic test produced locally would be desirable [7]. Third, doxycycline remains the standard treatment for SFG and TG rickettsioses [9]. Given that rickettsioses are endemic in Malaysia, it would be more than appropriate to have intravenous doxycycline available for treatments where patients are acutely ill. Although meropenem has not been commonly used to treat rickettsioses [9], it proved useful in this case and should be considered for this type of disease in Malaysia.

To conclude, there should be an emphasis on clinicians in Sabah to closely monitor patients with acute febrile illness given the historical and current prevalence of rickettsioses and the rapid and unpredictable fatal course of spotted typhus. Special attention must be given to those patients with glucose-6-phosphate dehydrogenase (G6PD) deficiency, a condition present in five to ten percent of all ethnic races in Sabah [34,35], since rapid fulminant courses of SFGR is known to occur in patients with this condition [36]. Therefore, typhus and typhus-like diseases must be included in the early differential diagnosis of nonspecific fevers in Sabah. In addition, a culture of preserving acute and convalescent samples for repetition of tests to confirm diagnoses must also be encouraged. Finally, without a rapid and reliable diagnostic test for confirming or excluding the disease at an early stage, the prompt administration of empirical antibiotic therapy to patients presenting symptoms related to rickettsioses is recommended in a closely-supervised manner to prevent antibiotic resistance, which will be detrimental at the individual and community level [8,37].

## Figures and Tables

**Table 1:** Laboratory test results, showing thrombocytopenia and transaminitis that persisted for several days post-admission.

Test	D1 *	D3	D5	D7	D13	D15	Reference Range
Haemoglobin, g/L	156	132	132	125	140	**129**	130–180
White blood cells, cells/L	4.6	**3.8**	6.6	10.1	7.6	5.8	4.0–11.0 × 10^9^
Lymphocytes, cells/L	**0.6**	**0.4**	2.1	**6.7**	**5.5**	**4.1**	1.5–4.0 × 10^9^
Platelets, cells/L	**62**	**38**	**106**	204	**408**	374	140–400 × 10^9^
Albumin, g/L	39	**30**	**24**	**26**	38	38	35–52
Bilirubin, µmol/L	**26**	16	18	9	12	10	<21
Alkaline phosphatase, U/L	84	74	**125**	**136**	114	100	30–120
Gamma-glutamyl transferase, U/L	**147**	**119**	**164**	**159**	**136**	**114**	<50
Aspartate transferase, U/L	37	43	**206**	**88**	38	33	<45
Alanine transaminase, U/L	35	36	**154**	**112**	**68**	54	<55
C-reactive protein, mg/L	N/T	N/T	**93.99**	**35.34**	2.55	N/T	>=5

* Admission day. N/T—not screened that day. Out-of-range values are highlighted in bold.
